# Supplementary material for: Reconciling Mining with the Conservation of Cave Biodiversity: A Quantitative Baseline to Help Establish Conservation Priorities
Source: PLoS One. 2016 Dec 20;11(12):e0168348. doi: 10.1371/journal.pone.0168348 (PMC5173368; doi:10.1371/journal.pone.0168348)
Supplement: S1 Dataset — (ZIP) [file pone.0168348.s002.zip › Taxa/Serra Norte/SN_2013/N4E/Lista N4E-35.pdf]

## CAVIDADE N4E-0035

| Classe | Ordem | Fam/Outros | Gên/Outros | Espécie | S-U | U-S | Zon. |
|---|---|---|---|---|---|---|---|
| Arachnida | Acari | Mesostigmata | | jovem | | X | E |
| Arachnida | Acari | Smarididae | | sp.19 | X | | E |
| Arachnida | Acari | Trombidiidae | Microtrombidiidae | sp. | | X | E |
| Arachnida | Araneae | Araneidae | *Alpaida* | sp.1 | X | X | E |
| Arachnida | Araneae | Ctenidae | | jovem | 1 | | E |
| Arachnida | Araneae | Drymusidae | *Drymusa* | jovem | | X | E |
| Arachnida | Araneae | Filistatidae | | sp.1 | X | | E |
| Arachnida | Araneae | Oonopidae | *Neoxyphinus* | sp.1 | X | | E |
| Arachnida | Araneae | Pholcidae | *Mesabolivar* | sp.1 | X | X | E |
| Arachnida | Araneae | Scytodidae | *Scytodes* | sp. | | X | E |
| Arachnida | Araneae | Segestriidae | *Ariadna* | sp.1 | X | | E |
| Arachnida | Araneae | Theridiidae | *Theridion* | sp. | X | X | E |
| Arachnida | Araneae | Theridiosomatidae | *Plato* | sp. | | X | E |
| Arachnida | Araneae | Uloboridae | *Uloborus* | sp. | | X | E |
| Arachnida | Opiliones | Manaosbiidae | *Saramacia* | sp.1 | 1 | | E |
| Arachnida | Opiliones | Stygnidae | *Protimesius* | *laevis* | 2 | | E |
| Arachnida | Palpigradi | | | jovem | | X | E |
| Arachnida | Pseudoscorpiones | Chernetidae | | sp. | | X | E |
| Chilopoda | Scutigeromorpha | Pselliodidae | | jovem | 1 | | E |
| Entognatha | Collembola | Entomobryidae | | sp.7 | X | | E |
| Insecta | Blattodea | Blattidae | | jovem | | X | E |
| Insecta | Coleoptera | Carabidae | | sp. | | X | E |
| Insecta | Diptera | Cecidomyiidae | | sp. | | X | E |
| Insecta | Diptera | Chironomidae | | sp. | X | | E |
| Insecta | Diptera | Dolichopodidae | | sp. | X | | E |
| Insecta | Diptera | Mycetophilidae | | sp. | | X | E |
| Insecta | Diptera | Psychodidae | *Psychodopygus* | *wellcomei* | X | X | E |
| Insecta | Diptera | Tipulidae | Tipulinae | sp. | X | | E |
| Insecta | Heteroptera | Reduviidae | Reduviinae | sp. | 1 | | E |
| Insecta | Homoptera | Cixiidae | | sp.3 | X | X | E |
| Insecta | Hymenoptera | Formicidae | *Brachymyrmex* | sp.1 | X | | E |
| Insecta | Hymenoptera | Formicidae | *Camponotus* | sp.1 | X | X | E |
| Insecta | Hymenoptera | Formicidae | *Dolichoderus* | aff. *bispinosus* | X | | E |
| Insecta | Hymenoptera | Formicidae | *Eciton* | sp. | | X | E |
| Insecta | Hymenoptera | Formicidae | *Gnamptogenys* | sp.1 | X | | E |
| Insecta | Hymenoptera | Formicidae | *Odontomachus* | *haematodus* | | X | E |
| Insecta | Hymenoptera | Formicidae | *Pachycondyla* | sp.2 | X | | E |
| Insecta | Isoptera | Termitidae | | sp. | | X | E |
| Insecta | Lepidoptera | Tineoidea | | jovem | | X | E |
| Insecta | Neuroptera | Myrmeleontidae | | jovem | | X | E |
| Insecta | Orthoptera | Phalangopsidae | *Phalangopsis* | sp. | 3 | X | E |
| Amphibia | Anura | Hylidae | | sp. | | 1 | E |
